# The Pathophysiological Underpinnings of Gamma-Band Alterations in Psychiatric Disorders

**DOI:** 10.3390/life14050578

**Published:** 2024-04-30

**Authors:** Annalisa Palmisano, Siddhartha Pandit, Carmelo L. Smeralda, Ilya Demchenko, Simone Rossi, Lorella Battelli, Davide Rivolta, Venkat Bhat, Emiliano Santarnecchi

**Affiliations:** 1 Chair of Lifespan Developmental Neuroscience, Faculty of Psychology, TUD Dresden University of Technology, 01069 Dresden, Germany; 2 Precision Neuroscience and Neuromodulation Program, Gordon Center for Medical Imaging, Massachusetts General Hospital, Harvard Medical School, Boston, MA 02115, USAesantarnecchi@mgh.harvard.edu (E.S.); 3 Department of Education, Psychology, and Communication, University of Bari Aldo Moro, 70121 Bari, Italy; davide.rivolta@uniba.it; 4 Siena Brain Investigation and Neuromodulation (SI-BIN) Laboratory, Department of Medicine, Surgery and Neuroscience, Neurology and Clinical Neurophysiology Section, University of Siena, 53100 Siena, Italy; simone.rossi@unisi.it; 5 Interventional Psychiatry Program, St. Michael’s Hospital—Unity Health Toronto, Toronto, ON M5B 1W8, Canada; ilya.demchenko@unityhealth.to (I.D.);; 6 Institute of Medical Science, Temerty Faculty of Medicine, University of Toronto, Toronto, ON M5S 1A1, Canada; 7 Berenson-Allen Center for Noninvasive Brain Stimulation, Department of Neurology, Beth Israel Deaconess Medical Center, Harvard Medical School, Boston, MA 02215, USA; 8 Center for Neuroscience and Cognitive Systems@UniTn, Istituto Italiano di Tecnologia, 38068 Rovereto, Italy; 9 Department of Psychiatry, Temerty Faculty of Medicine, University of Toronto, Toronto, ON M5S 1A1, Canada; 10 Department of Neurology and Radiology, Massachusetts General Hospital, Boston, MA 02114, USA

**Keywords:** gamma-band oscillations, parvalbumin-positive interneurons, perineuronal nets, schizophrenia, bipolar disorder, major depressive disorder

## Abstract

Investigating the biophysiological substrates of psychiatric illnesses is of great interest to our understanding of disorders’ etiology, the identification of reliable biomarkers, and potential new therapeutic avenues. Schizophrenia represents a consolidated model of γ alterations arising from the aberrant activity of parvalbumin-positive GABAergic interneurons, whose dysfunction is associated with perineuronal net impairment and neuroinflammation. This model of pathogenesis is supported by molecular, cellular, and functional evidence. Proof for alterations of γ oscillations and their underlying mechanisms has also been reported in bipolar disorder and represents an emerging topic for major depressive disorder. Although evidence from animal models needs to be further elucidated in humans, the pathophysiology of γ-band alteration represents a common denominator for different neuropsychiatric disorders. The purpose of this narrative review is to outline a framework of converging results in psychiatric conditions characterized by γ abnormality, from neurochemical dysfunction to alterations in brain rhythms.

## 1. Introduction

Electric current generation within the brain results from multiple sources (i.e., synaptic activity, intra- and extracellular ionic fluxes through voltage-gated and ligand-gated channels, and intrinsic membrane potential resonance) [1,2]. Extracellular electric fields’ spatiotemporal summation gives rise to neural oscillations, which are considered to reflect neuronal populations’ activity [3]. It is still debated whether neural oscillations are merely an epiphenomenon of the coordinated neuronal activity or actively contribute to state-dependent informational inflow, outflow, and integration across different brain regions [4]. Nevertheless, brain rhythms hold functional significance as they arise from brain dynamics underlying cognitive processes [5]. Consistent with this, neural oscillations’ distinct frequency components have been associated with unique brain functions and states [6], ranging from basic sensory processes to higher-order cognitive operations [7,8,9,10].

Among the spectrum of the characteristic frequency bands, gamma (γ) oscillations (30–80 Hz) have been related to higher brain functions (e.g., the attentive processing of information and active maintenance of memory contents) [11,12,13]. Disturbances in γ rhythms have been reported in multiple neuropsychiatric conditions [2,14,15], even prior to symptom onset [16]. Schizophrenia (ScZ) represents a prime example of brain rhythm disruption, with γ oscillations recognized as a core substrate of clinical symptoms and cognitive deficits [17]. Similar evidence has been reported for bipolar disorder (BD) and, less robustly, major depressive disorder (MDD).

Gamma-band alterations are hypothesized to result from altered cortical excitatory/inhibitory (E/I) balance [18,19,20,21,22]. The subsequent shift towards epileptic hyperexcitability [22,23] has been found to characterize multiple neurodevelopmental [24], neurodegenerative [25], and psychiatric diseases [26]. The proper functioning and stability of brain circuits are based on the temporal balance between excitatory and inhibitory inputs from glutamatergic pyramidal cells and inhibitory GABAergic interneurons, respectively [27], with intracortical networks of specialized GABAergic interneurons (i.e., with fast, strong, and shunting inhibitory synapses) orchestrating the generation of fast oscillations [28]. The functional assessment of the E/I imbalance (e.g., via the aperiodic 1/f-like component of the neural power spectrum) shows an association between a shift towards excitation and a decrease in high-frequency activity oscillations [29,30,31]. E/I imbalance caused by a predominant GABAegic dysfunction has been proposed as a unifying hallmark of different neuropsychiatric diseases [32,33,34,35,36,37].

At the cellular level, γ oscillations are thought to arise from the activity of parvalbumin-positive (PV+) cells, a subpopulation of GABAergic interneurons exhibiting fast-spiking (FS) properties [38,39,40], with in vitro and in vivo studies showing the firing of PV+ cells to be robustly coupled to γ oscillations [40,41,42,43,44]. Critical for this purpose are α1-subunit-containing GABAA receptors, which display fast kinetics and are abundant in PV+ interneuron synapses [45,46]. PV+ interneuron maturation is crucial for the onset of critical periods (i.e., time windows of enhanced plasticity) [47]. Indeed, a delayed or extended period of circuit instability in the developing brain—that is, the E/I imbalance—is consistent with a loss of PV+ function in psychiatric disorders [48].

**Figure 1:**
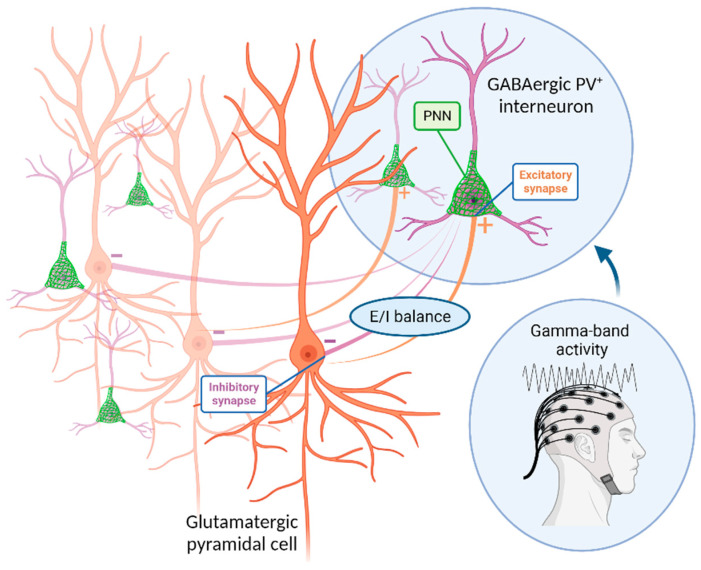
Schematic representation of the interaction between GABAergic PV+ interneurons and pyramidal neurons. Pyramidal neurons send excitatory (+) glutamate-mediated inputs to fast-spiking PV+ interneurons, which in turn send inhibitory (−) feedback signals back to the pyramidal cells. Activated interneurons can propagate inhibitory long-range signals to multiple pyramidal cells and synchronize their activity (E-I balance). PV+ = parvalbumin-positive, E = excitatory, I = inhibitory, PNN = perineuronal net. Created in BioRender.com.

Interneuronal dysfunction has been associated with the impaired or delayed development of the perineuronal nets (PNNs), the predominant extracellular matrix (ECM) aggregates responsible for neuronal structural support, synaptic functions, and plasticity [49,50,51,52,53]. Immunolabeling evidence with markers for PNNs’ chondroitin sulfate proteoglycans (CSPGs) shows that PNNs mainly encompass GABAergic PV+ cells [54,55,56,57] and regulate FS firing [58,59]. The behavioral or in vitro manipulation of neuronal activity during critical periods has been shown to reduce the PNNs, which suggests that their expression is activity-dependent and tied to both the development of PV+ neurons and the establishment of the E/I balance in neural circuits [60,61,62] (Figure 1).

Oxidative stress and neuroinflammation are thought to affect the structural and functional integrity of PNNs by mediating excessive microglia activation and the release of ECM-cleaving enzymes, such as matrix metalloproteinases (MMPs) [63,64]. Research on PNN modulators in psychiatric conditions showed that abnormal rates of PNN-degrading proteases, such as metalloproteinase-9 (MMP-9) [65], or a diminished expression of reelin (i.e., a key protein of the PNNs expressed in GABAergic neurons and secreted into PNNs [66,67]) negatively impact PV+ cell-mediated activity [68,69,70,71,72]. Excessive PNN cleavage, in turn, leads to the formation of reactive oxygen species (ROS), exacerbating microglia activation and neuroinflammation [73,74]. Evidence from animal models points to PNN abnormalities as the main source of antioxidant defence failure [75], with subsequent cortical alterations attributable to the loss of PV+ interneurons [58,76,77].

The outlined evidence delineates a pathophysiological cascade of abnormal γ rhythms in various neuropsychiatric conditions resulting from abnormalities in PV+ interneurons, the disruption of PNNs, and neuroinflammation. Despite not constituting a complete etiological explanation, this model of pathogenesis is supported by converging molecular, cellular, and functional evidence.

Worth noting, ScZ, BD, and MDD are quite complex conditions that result from the interplay of various etiological factors such as genetic predisposition [78,79], environmental influences [80,81], neurotransmitter dysregulation [82,83,84], and structural brain abnormalities [85,86,87]. The purpose of the current narrative review is to provide a pathophysiological framework of γ-band disturbances and the underlying mechanisms in ScZ, BD, and MDD, with evidence of alterations from the micro- (i.e., cellular, molecular) to the macro-scale (i.e., brain oscillations).

## 2. Schizophrenia-Related Pathophysiological Cascade

ScZ represents the most common and complex form of functional psychosis and the seventh-most-costly medical illness in our society, affecting about 0.3 percent of the population worldwide [88,89]. It is characterized by a combination of symptoms classified into ‘positive’ (e.g., delusions, hallucinations) and ‘negative’ (e.g., social withdrawal, apathy, impaired judgment) [90]. The etiology and pathogenesis remain unclear due, in part, to the heterogeneity of the ScZ phenotype and the lack of clear pathological markers such as those that provide reference points in the study of other neuropsychiatric disorders [91,92].

### 2.1. Neuroinflammation and Redox Dysregulation in Schizophrenia

One hypothesis for ScZ etiology is that perinatal infections (e.g., due to spirochetes such as *Borrelia burgdorferi*, and viruses such as Epstein–Barr virus) might trigger a neuroinflammatory response that persists during adulthood [93,94,95,96,97]. Indeed, a growing body of evidence has revealed signs of systemic and central nervous system inflammation in ScZ patients [98,99,100,101], such as an increase in inflammatory markers (e.g., neutrophil-lymphocyte ratio, proinflammatory cytokines) [96,102] and evidence for microglial (i.e., the brain macrophage) overactivation [103,104,105]. The inflammatory overproduction of ROS increases cellular oxidative stress, leading to neurotoxic and detrimental effects on brain development and the maturation of PV+ neurons [106,107].

Excessive oxidative stress directly affects PNNs by overstepping their protective capacity, which in turn impacts PV+ cells [108,109]. Given their high energy demand [110], the maturation of PV+ interneurons and their proper functioning require a well-regulated antioxidant system and seem to be particularly vulnerable to ROS overproduction [111,112]. Indeed, early-life inflammatory conditions cause a persistent decrease in hippocampal and prefrontal cortical PV+ cells in animal models [113,114] and determine susceptibility to ScZ [115,116,117]. Consistently, post-mortem studies have reported increased oxidative stress in the prefrontal cortex, anterior cingulate cortex, caudate region, and hippocampus in subtypes of ScZ [118,119,120,121,122].

### 2.2. Perineuronal Net Abnormalities in Schizophrenia

PNN alterations have been solidly implicated in the pathophysiology of ScZ [57]. Post-mortem studies reveal alterations in PNN structure and biological/biochemical composition in the deep amygdala nuclei and entorhinal cortex [123,124,125]. The evidence for massive CSPG abnormalities (i.e., the main components of mature PNNs) in glial cells further supports the pivotal role of ECM–glial interactions in the pathogenesis of this disease [126]. Consistently, extracellular analyses of the cortex of ScZ patients show significant alterations, such as water diffusion anisotropy in tissue, together with markers for neuroinflammation and microglia activation [127]. Noteworthy, the *PTPRZ1* gene encoding for the CSPG *phosphacan* has been implicated in susceptibility for ScZ [128], as well as growth factors affecting astrocytic CSPG expression [129].

ECM alterations in the brain tissue of ScZ patients have also been detected in the neocortex, with evidence for reduced PNN density in layers three and five of the PFC [130]. Despite Enwright and colleagues [131] reporting normal PNN density in the dorsolateral prefrontal cortex (DLPFC), the fluorescence intensity of *Wisteria floribunda*-Agglutinin (WFA) labeling and immunohistochemical staining for *aggrecan* (i.e., the primary CSPG of PNNs) was lower around PV+ interneurons in ScZ brains. The role of PNNs in prefrontal regions comprises the regulation of the synaptic refinement that occurs through the peripubertal period until late adolescence and early adulthood, with its dysfunction compromising the experience-dependent consolidation of synaptic connectivities [132,133,134]. This cascade of effects temporally matches with the usual age of onset for ScZ symptomatology, thus providing further support for the hypothesis that PNN abnormalities primarily affecting PV+ cells exert a core role in the impairment of cortical integrity and stability in ScZ [135,136].

### 2.3. PV+ Interneuron Alterations in Schizophrenia

Alterations in PV+ interneurons have been consistently implicated in the pathophysiology of ScZ, with evidence pointing towards altered GABA synthesis, abnormal transmission, and structural abnormalities [137,138,139]. Proof for GABAergic inhibitory circuitry alterations in ScZ mainly derives from molecular evidence on gene expression in the PFC—for instance, investigating the mRNA expression of GAD67, a GABA-synthesizing enzyme [140,141]. Indeed, Hashimoto and colleagues [140] found reduced PV expression in the brain of ScZ patients, which was correlated with a decrease in the density of neurons containing GAD67 mRNA. The co-localization of the reduction in PV- and GAD67-mRNA expression supports the idea that GABA neurotransmission mediated by PV+ interneurons is altered in ScZ patients [142].

Multiple studies also revealed abnormalities involving GABAA postsynaptic receptors containing the α1 subunit, which is the class of GABA receptors exhibiting the fastest kinetics [45,143]. Lower expression of the mRNA for the α1 subunit has been detected in the neocortex, particularly in layers three and four of the PFC of ScZ patients [144,145]. This specific molecular deficit results in the reduced density and strength of PV+ inhibitory inputs to the pyramidal cells [146]. An increase in the mRNA for the GABAA receptor α2 subunits has also been detected in the PFC of patients with ScZ and interpreted as a compensatory response to the deficient GABA activity and release due to lower levels of GAD67 [144,147]. The lower density of the GABA membrane transporter GAT-1 has also been found in patients’ PFC [148], suggesting a reduced reuptake of this neurotransmitter. This might be linked to the altered inputs from the medial dorsal thalamus, which are critical for initiating and maintaining γ activity [149,150].

Reduced density of hippocampal PV+ interneurons has been reported in mice lacking the *tyrosine kinase ErbB4* receptor for Neuregulin-1 (*NRG1*), whose expression is highly enriched in both the chandelier and basket subclasses of PV+ cells in the PFC, hippocampus, and amygdala [151]. Both *ErbB4* and *NRG1* are susceptibility genes for ScZ [152]. Interestingly, PV+ cell alteration in this model is associated with impaired network inhibition and reduced γ oscillatory power [153]. In vivo, the loss of *NRG1/ErbB4* signaling in the developing brain causes a reduction in the number of GABAergic interneurons in the postnatal cortex [154], and inhibition of *NRG1/ErbB4* signaling in PV+ interneurons of the *PV-ErbB4−/− mice* is associated with reduced GABAergic activity and the E/I imbalance [155]. Zhang and Reynold found the relative densities of hippocampal PV-immunoreactive cells in post-mortem brains to be deficient in ScZ [156]. This evidence can be linked to the role of PV+ interneurons in the generation of hippocampal oscillations [157,158]. Moreover, this reduction was unrelated to antipsychotic drug treatment, age, or duration of illness.

Mice expressing a truncated version of the ScZ risk gene *DISC1* have reduced PV immunoreactivity in the PFC and hippocampus [159,160]. Interestingly, the *DISC1* pathway is convergent with those of other disease-related genes, including *NRG1* and *β*-amyloid precursor protein (APP) during cortical development [161]. Even though beyond the scope of this review, it is worth noting that APP gene mutation is associated with an early onset familiar form of Alzheimer’s disease [162], a neurodegenerative disease in which PV+ dysfunction and brain rhythms disturbances have been widely described [37,163]. 

Despite results on the relative density of PV+ cells in ScZ not always being consistent (e.g., Glausier and colleagues reporting lower PV protein levels and unchanged density of cell inputs [141]), the extensive evidence from molecular studies corroborates the core role of GABAergic PV+ interneuron dysfunction in the pathophysiology of ScZ.

### 2.4. Gamma-Band Oscillatory Alterations in Schizophrenia

The disturbance of neural oscillations in the γ-band is considered a core pathophysiological feature of ScZ, particularly in the generation of cognitive dysfunction [164,165]. Multiple animal models of ScZ support disturbances in γ oscillations—for instance, in terms of abnormalities in the rhythmic network activity, as well as proneness to hippocampal epileptiform activity [153,166]. Lodge and colleagues [167] showed that a decreased density of PV+ interneurons throughout the medial prefrontal cortex (mPFC) and ventral subiculum correlates with a reduced γ-band response to a conditioned tone during a latent inhibition paradigm. Similarly, Nguyen and colleagues [168] found that the inhibition of PV+ or GAD65 neurons alters network oscillatory activity in the ventral hippocampus (vHPC) of rats and generates sensorimotor deficits [169]. The transgenic mouse in the study from Cho and colleagues [170] (i.e., impaired *Dlx5* and *Dlx6* transcription factors involved in the development of PV+ interneurons) exhibited a reduction in evoked γ-band oscillations, as well as cognitive inflexibility. Notably, optogenetic stimulation of cortical PV+ neurons in the γ frequency (40Hz) was capable of restoring cognitive flexibility. In an animal model of first-episode psychosis, spatial memory deficits were accompanied by disturbances in hippocampal θ-γ oscillations (i.e., reduction, uncoupling) and long-term potentiation mechanisms [171].

Alterations of γ-band activity in ScZ patients have been demonstrated with different experimental paradigms (i.e., spontaneous and evoked activity/responses). For instance, Grent-’t-Jong and colleagues [172] found individuals meeting clinical high-risk criteria for ScZ to exhibit increased γ power at rest, while both first-episode and chronic patients showed aberrant spectral power at both low and high γ-band frequencies. Moreover, the shift in E/I-balance toward augmented excitation found in high-risk subjects correlated with increased occipital γ power. The differential oscillatory patterns across illness stages are of great interest in the field of biomarkers research. For instance, increased excitation and resting-state γ disturbances might constitute spectral markers to predict the onset of psychosis [173]. Consistent with this, both patients and their unaffected siblings were found to exhibit reduced γ activity in the parietal cortex relative to non-affected individuals [174]. Also, impaired perceptual integration and disorganized symptoms in ScZ are accompanied by a pronounced reduction in spectral power in the γ-band [175]. The sub-anesthetic administration of ketamine (i.e., N-methyl-D-aspartate receptor (NMDA-R) antagonist leading to psychopathology as observed in ScZ) in healthy volunteers was found to induce dysregulated γ activity in thalamo-cortical networks [172,176].

In addition to abnormalities in spontaneous oscillations, altered stimuli-evoked γ responses have been reported with both visual [177] and auditory paradigms [178]. An EEG-fMRI study demonstrated a γ-band-specific network (involving bilateral auditory cortices, thalamus, and frontal brain regions) to be less active in subjects at high risk for ScZ, together with worse task performance [179]. This has been linked to a specific dysfunction in the frontotemporal network responsible for γ-phase synchronization and indexed by the ‘gamma-band auditory steady-state response’ (ASSR), which represents a robust biomarker that is increasingly studied in neuropsychiatric disorders as a reflection of deficient γ-band oscillations [19,180]. Abnormal ASSRs have been reported in first-episode and chronic ScZ patients, with specific frequency patterns associated with the severity of hallucinatory experiences [181,182]. Also, visually induced γ-band activity has been shown to correlate with genetic risk for ScZ [183]. This further strengthens the idea of neuronal firing-pattern dysfunction as a pathophysiological factor, rather than a consequence, in the development of this disorder.

The literature investigating oscillatory brain networks in ScZ points towards alterations in both local and long-range oscillatory network dynamics [175,176,184]. As such, patients with ScZ exhibit spatial functional hyper- or disconnectivity [174], as well as disturbances in the temporal structure of γ-band oscillations [185,186] (e.g., abnormalities in the Default Mode Network [DMN] have been reported and associated with decreased coherence in the γ-band at rest [187,188]). Transcranial magnetic stimulation (TMS), which activates cortical neurons via time-varying magnetic fields eliciting action potentials, can be coupled with electroencephalography (EEG) to investigate cortical γ modulation in ScZ [189]. Ferrarelli and colleagues [190] found that ScZ patients exhibit decreased EEG-evoked responses in the γ-band when TMS is applied over the frontal cortex relative to healthy controls. Also, repetitive TMS (rTMS) has been applied over the DLPFC to normalize γ oscillations and cognitive performance in patients [32]. Indeed, an extensive body of research has adopted neuroimaging approaches such as multimodal non-invasive brain stimulation techniques (NIBS) to investigate the neurophysiological correlates of abnormal brain activity, as well as for interventional purposes. Promising results emerge from the recent use of transcranial Alternating Current Stimulation (tACS) [191,192,193]. This technique allows for frequency-specific entrainment of endogenous neural oscillations and, potentially, induction of long-term synaptic plasticity [194]. When applied at the γ frequency over prefrontal areas, it has been shown to improve positive and negative symptoms, cognitive status, and well-being in ScZ patients [195].

**Figure 2:**
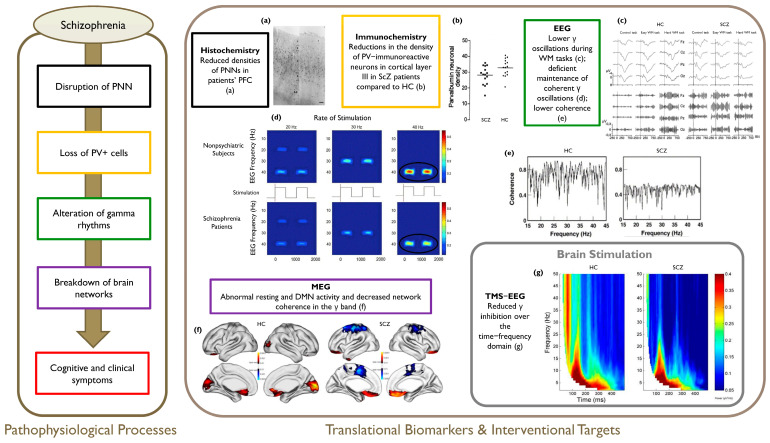
Overview of the outlined pathophysiological cascade in ScZ (**left panel**) with relevant sample evidence (**right panel**). (**a**) Representative photomicrographs showing reduced density of PNNs in the PFC of patients with ScZ as compared to HC (Reprinted from [130] with permission from Elsevier); (**b**) Scatterplots showing lower cortical density of PV+ interneurons in the DLPFC of SCZ patients vs. HC (horizontal lines indicate mean values) (Reprinted from [196] with permission from Elsevier); (**c**) γ rhythms abnormalities during WM tasks in ScZ patients vs. HC: grand average event-related oscillations in HC (**left upper panel**) and ScZ patients (**right upper panel**) during N-back tasks with varying WM demands (easy/hard), with T = 0 ms representing the stimulus onset (Reprinted from [197] with permission from Elsevier); (**d**) Intertrial coherence time/frequency analyses: 40-Hz phase-locking deficits in ScZ patients (**lower panel**) relative to HC (**upper panel**) (Reprinted from [198] with permission from Elsevier); (**e**) Decreased coherence in higher frequency ranges (β and γ) between central and frontal areas in ScZ patients vs. HC (Reprinted from [199] with permission from Elsevier); (**f**) Gamma-power difference maps between resting state and cognitive task in HCs and ScZ patients (Reprinted from [188] (licensed under CCBY 4.0, https://creativecommons.org/licenses/by/4.0/, accessed on 7 December 2023)); (**g**) Reduced frontal inhibition in ScZ patients compared to HCs following DLPFC stimulation (Reprinted from [200] (licensed under CCBY 4.0, https://creativecommons.org/licenses/by/4.0/, accessed on 7 December 2023)). PNN = Perineuronal Net; PFC = Prefrontal Cortex; HC = Healthy Controls; PV+ = Parvalbumin-positive; DLPFC = Dorsolateral Prefrontal Cortex; WM = Working Memory; EEG = Electroencephalography; DMN = Default Mode Network; TMS = Transcranial Magnetic Stimulation; ERSP = Event-Related Spectral Perturbation.

A complex clinical phenotype arises from the described pathophysiological scenario. We believe that the study of γ brain rhythms and the underlying molecular and cellular mechanisms might provide a bridge between the neural and psychopathological levels. See Figure 2 for an overview of the outlined framework in ScZ.

## 3. Bipolar Disorder-Related Pathophysiological Cascade

BD is a recurrent chronic disorder affecting 1% of the world’s population [201]. Patients experience episodes of depression (i.e., low mood and related symptoms) and episodes of either mania (i.e., elated or irritable mood, or both) or hypomania (i.e., less severe or less-protracted symptoms of mania) [202]. Based on the extent and severity of mood elevations, BD is classified along a spectrum from unipolar to bipolar II or bipolar I [203]. Given the high rate of clinical comorbidities and the complexity of diagnosis, a further understanding of the underlying pathogenesis would support the development of reliable biomarkers for diagnosis and treatment [204,205].

### 3.1. Neuroinflammation and Redox Dysregulation in Bipolar Disorder

In the last decade, several studies have evaluated whether inflammatory and redox dysregulation processes might be involved in the pathophysiology of BD [206]. As reported for ScZ, prenatal exposure to infectious or inflammatory insults might contribute to the etiology of BD. As such, studies on animal models of prenatal maternal immune activation (MIA) showed that immune activation leads to the transgenerational transmission of pathological traits and behavioral alterations [207,208]. Mice with abnormal prefrontal levels of glutathione—the major cellular redox regulator and antioxidant—exhibit behavioral, morphological, electrophysiological, and neurochemical alterations analogous to those observed in patients. At the neuronal level, redox imbalance causes the impairment of PV+ interneurons and abnormal neuronal synchronization [209]. Increased concentrations of neuroinflammatory markers (e.g., Interleukin (IL)-1β, IL-6, IL-10) have been reported in the frontal cortex and hippocampus of a mouse model of mania [210].

Mitochondrial dysfunction has been extensively detected in BD patients [121,211,212] and linked to high levels of oxygen consumption and ROS overproduction [213]. Oxidative stress resulting from dysfunctional mitochondria leads to metabolic disturbances in neurons and glia, cell damage, and further oxidative stress, stimulating the generation of proinflammatory cytokines [206,214]. Proof for peripheral proinflammatory status (e.g., increased Tumor Necrosis Factor-α, TNF-α) in patients during mania [215] and its link with other markers of BD (e.g., abnormalities in circadian rhythmicity, metabolism) suggest that inflammatory mechanisms play a crucial role in the pathophysiology of this disease.

### 3.2. Perineuronal Net Abnormalities in Bipolar Disorder

Various lines of evidence support ECM abnormalities in BD. A post-mortem study by Knable and colleagues reported decreased reelin together with PV+ cell reduction in the brains of BD patients [216]. Further evidence revealed reduced glial cell immunoreactivity to the PNN’s CSPG *aggrecan* in the amygdala of BD patients compared to healthy subjects, together with lower PNN clusters [124]. Indeed, structural changes in BD brains have been detected in multiple amygdala nuclei in terms of volume, neuronal density, and size [217]. Steullet and colleagues [218] found a decrease in PNNs in the thalamic reticular nucleus of BD brains, with no effects on the duration of illness or the age of onset, together with a decrease in the number of PV+ cells and altered neuronal activity. Similar results emerged in transgenic mice with redox dysregulation compared with the wild-type mice [218]. A lower density of PNNs was also detected in the DLPFC of BD patients [219]. Despite the outlined converging evidence on PNN contribution to disease onset, the literature also shows inconsistencies [130]. Arguably, the differences could be quantitative rather than qualitative, and deficits could be region-specific or based on disease-specific timelines.

A specific mechanism of ECM molecular alterations in BD involves PNN regulation by homeoprotein *Otx2*, which controls the onset and closure of critical periods by triggering PNN expression and maturation [220]. *Otx2* accumulation in PV+ cells via PNN interaction is required to maintain the non-plastic state of the adult cerebral cortex [221]. Notably, polymorphisms in *Otx2* have been associated with BD and might be implicated in alterations of PNN-PV+ assembly dynamics in this disease [222]. The impact of *Otx2* dysregulation on PNN formation might be amplified by redox dysregulation: decreased PNN formation due to redox imbalance prevents the internalization of *Otx2* by PV+ cells, which in turn leads to a reduction of the PNN staining [223,224].

Genomic evidence supports the association of the ScZ risk gene Neurocan (*NCAN*) and specific mania symptoms in BD [225,226], supporting a genotype overlap between BD and ScZ [227]. Importantly, Neurocan is one of the main ECM proteoglycans mediating both the proper formation of PNNs and its proteins during brain development and the amount of GAD65/67 in GABAergic synapses [228].

### 3.3. PV+ Interneuron Alterations in Bipolar Disorder

As for the involvement of PV+ interneurons in BD, animal models of ankyrin-G isoform imbalance affecting PV+ cells exhibit a lack of axonal voltage-gated sodium channels, altered PV+ firing thresholds, and a diminished action-potential dynamic range [229]. Notably, there were behavioral changes in a mice model of BD patients. Evidence from post-mortem studies demonstrates the GAD65/67 enzymes for GABA synthesis are downregulated in the cerebellum and hippocampus of BD subjects compared to controls [230,231]. Guidotti and colleagues [66] also reported a reduced PFC and cerebellar expression of both GAD67 protein and GAD mRNA. Remarkably, the authors found a decreased expression of reelin, supporting the relevance of both GABAergic and ECM abnormalities in BD.

Fatemi and colleagues [232] also reported protein reduction for both the GABBR1 and GABBR2 subunits of the GABAB receptor in the lateral cerebella of BD brains (as in ScZ and MDD). As such, GABAB receptors are responsible for presynaptic and postsynaptic inhibition in PV+ interneurons and contribute to the dynamic modulation of their activity during oscillations [233]. Another study demonstrated the GABABR alterations in the PFC of BD subjects to be similar to those in ScZ [234]. Further neurochemical evidence consists of a reduced cortical concentration of GABA in the occipital cortex of recovered BD patients [235]. Moreover, low GABA and GAD concentrations have been reported in patients’ cerebrospinal fluid and plasma, respectively [236,237]. Disconfirmations in the detection of low GABA levels appear to be marginal and related to the limitations in techniques (e.g., regional analysis through magnetic resonance spectroscopy [MRS] resulting in high measurement variance and low specificity) [238].

Despite fewer immunochemical and cytoarchitectural studies being available for PV+ interneuron alterations in BD compared to ScZ, abundant evidence reveals the reduced density of cortical GABAergic interneurons in the anterior cingulate cortex, PFC, and parahippocampal region of BD brains [239,240,241]. In addition to density, the spatial distribution of GABAergic interneurons was found to be altered [242]. Steullet and colleagues [218] tested the disruption of cortico-thalamo-cortical circuits in BD with a combination of human post-mortem and rodent studies on PV+ neurons: both transgenic mice and patients’ brains showed a decrease in the density of PV+ cells. Indeed, BD subjects exhibit significantly reduced PV mRNA in different cortical layers of the PFC [243]. Some studies revealed GABAergic and PV+ abnormalities in BD that are not detected in ScZ (e.g., the density of PV+ interneurons in the entorhinal cortex) [244]. Despite the fact that GABAergic deficits in BD have been found to generally match those in psychosis, they might differ in cortical localization (e.g., deficits in hippocampal PV+ are more profound in ScZ patients) [245] and severity [246].

### 3.4. Gamma-Band Oscillatory Alterations in Bipolar Disorder

Abnormalities in γ-frequency band activity are hypothesized to be integral to the pathophysiology of BD. Results from studies on mice models of maternal immune activation (MIA) (i.e., a psychiatric disease risk factor) provided evidence for a selectively reduced transmission in PV+ interneurons as well as for the disruption of γ activity in the mPFC, with concurrent behavioral alterations (i.e., anxiety) [207]. Prospective birth cohort studies highlight the associations between MIA and offspring risk of BD [247], as well as ScZ [248]. This also highlights the above-mentioned relevance of immune-related inflammatory mechanisms in the etiology of psychiatric disorders. Further evidence for γ abnormalities derives from the Clock-Δ19 mice, a model for alteration of the CLOCK gene conveying risk for BD. Specifically, the phasic entrainment of the nucleus accumbens (NAC) low γ (30–55 Hz) to delta (δ, 1–4 Hz) oscillations was found to be impaired in the Clock-Δ19 mice compared to wild-type mice [249]. Interestingly, the transgenic mice mania-like behavior ameliorated after the administration of lithium, a medication commonly used to manage similar behavioral manifestations in human patients suffering from BD [250].

Human studies show disturbances of synchronization and oscillations within brain networks measured with EEG at rest [251]. Specifically, changes in network characteristics for the γ-band were associated with the severity of patient symptoms. Özerdem and colleagues [252,253] investigated long-distance event-related γ coherence during a visual oddball paradigm with light stimulation in patients with BD. The authors reported disturbances in functional long-range connectivity as compared to healthy controls (e.g., patients’ lower coherence values in response to non-target stimuli). Moreover, γ oscillations were also investigated through auditory oddball and paired-click paradigms administered to BD patients, monozygotic BD twins, unaffected relatives, and healthy controls. Results showed reduced γ power in patients as compared to unaffected relatives [254]. Moreover, low and high γ ASSRs (that have been suggested to reflect the efficiency of inhibitory interneuronal activity) and ASSR γ synchronization were found to be reduced in BD patients [255,256,257]. Interestingly, induced and evoked γ responses to visual grating stimuli (i.e., stimuli known to induce primary visual γ oscillations) were investigated in subjects suffering from schizoaffective BD, a clinical syndrome with features of both BD and ScZ. Results showed that subjects with schizoaffective BD have increased visual cortical γ power as compared to controls [258]. Similarly to ScZ, the literature on BD investigating γ disturbances supports its role as a valuable candidate biomarker for this disorder [259].

The majority of studies adopting brain stimulation in BD investigate cortical inhibitory deficits (i.e., short-interval cortical inhibition [SICI], cortical silent period [SP], and interhemispheric inhibition [IHI]) [260,261], while the evidence for NIBS targeting γ oscillations is scarce. In a TMS-EEG study assessing event-related spectral perturbations (ERSPs), Canali and colleagues showed that TMS stimulation results in patients’ lower activation of γ activity in the frontal cortico-thalamo-cortical circuits as compared to healthy controls [262]. Interestingly, the same finding emerged for ScZ and MDD. TMS-EEG has also been adopted to assess brain oscillatory activity before and after antidepressant treatments. Canali et al. assessed ERSPs in TMS/EEG sessions before and after sleep deprivation and light therapy exposure, showing that patients exhibit lower activation of the frontal γ-band response compared to healthy participants. Furthermore, TMS stimulation did not result in changes in the γ-band response in patients, irrespectively of remission occurrence [263]. This could suggest that a reduced γ response to external perturbation in the PFC might represent an endophenotype, not modifiable with pharmacological therapy, of psychotic dysfunction [264].

**Figure 3:**
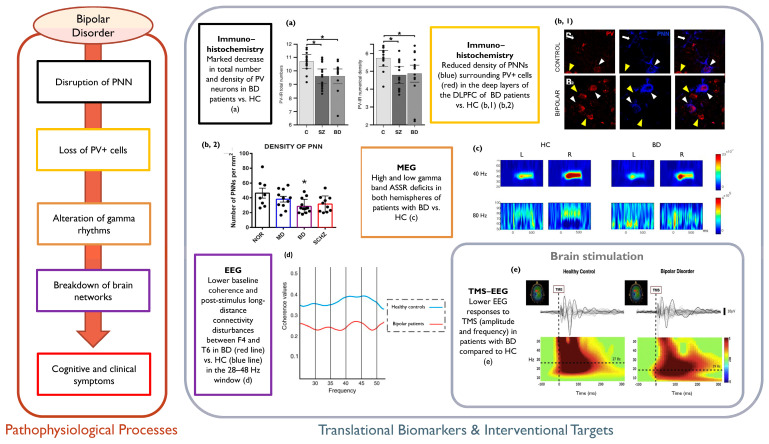
Overview of the outlined pathophysiological cascade in BD (**left panel**) with relevant sample evidence (**right panel**). (**a**) Decrease in PV+ neurons/PNNs number and density in the TRN of patients with BD as compared to HC (* indicate significance) (Reprinted from [218] (licensed under CCBY 4.0, https://creativecommons.org/licenses/by/4.0/, accessed on 7 December 2023); (**b**,**1**) Panoramic confocal microphotograph showing PNNs (blue) surrounding PV+ somata (red) in the deep layers of the DLPFC in HC vs. BD patients (white arrowheads: PV+ somata surrounded by PNNs, yellow arrowheads: PV+ cells lacking PNNs, white arrows: PNNs surrounding PV+ somata); (**b**,**2**) Histograms of significant differences in the density of PNNs in HC vs. BD patients (as well as SCZ and MDD) (* indicates significance) (Reprinted from [219] (licensed under CCBY 4.0, https://creativecommons.org/licenses/by/4.0/, accessed on 7 December 2023); (**c**) Group averaged time-frequency maps of ASSR power for each hemisphere, with color scales signifying ASSR power, showing BD patients’ reduced mean ASSR power and PLF to 40- and 80- Hz stimulation (Reprinted from [256] (licensed under CCBY 4.0, https://creativecommons.org/licenses/by/4.0/, accessed on 7 December 2023)); (**d**) Lower post-stimulus EEG coherence between F4 and T6 locations in BD patients (red line) as compared to HC (blue line) (Reprinted from [252] with permission from Elsevier); (**e**) Representative data from an HC and a BD patient: average EEG responses to TMS (grey traces represent the 60 recording channels) for the channel closest to the stimulation site (black trace) over the premotor area; color-coded: ERSP plots reflecting the significant TMS-related changes in amplitude and their duration (Reprinted from [263] with permission from Cambridge University Press). PV+ = Parvalbumin-positive; PNN = Perineuronal Net; TRN = Thalamic Reticular Nucleus; BPD = Bipolar Disorder; SCZ = Schizophrenia; HC = Healthy Controls; DLPFC = Dorsolateral Prefrontal Cortex; SCZ = Schizophrenia; ASSR = Auditory Steady-State Response; PLF = Phase-Locking Factor; EEG = Electroencephalography; TMS = Transcranial Magnetic Stimulation; ERSP = Event-Related Spectral Perturbation.

The outlined literature suggests that the pathophysiology of γ activity alterations might support the identification of novel markers of BD and the development of valuable therapeutic trajectories. See Figure 3 for an overview of the outlined framework in BD.

## 4. Major Depressive Disorder-Related Pathophysiological Cascade

Depression is an extremely common mood disorder accounting for the highest proportion of the global burden attributable to mental illness [265]. MDD, the most common type of depression, consists of a distinct collection of symptoms, including lowered mood, loss of interest or pleasure (anhedonia), and persistent feelings of guilt, as well as somatic disturbances such as impaired sleep, appetite, energy levels, and sexual functioning [266]. Even though several biological mechanisms with a possible role in MDD etiology and progression have been identified [267], a unified pathophysiology of the disease is still lacking.

### 4.1. Neuroinflammation and Redox Dysregulation in Major Depressive Disorder

Compared to ScZ and BD, no infectious insults seem to be involved in the onset of MDD [268]. However, neuroinflammation seems to participate in the genesis and development of this disorder (e.g., via ROS contribution to excessive MMPs activation perpetuating inflammation, and aberrant microglial activity [100,269,270,271]). Excessive inflammatory cytokines have been detected and linked to synaptic plasticity disruption and damage in MDD [272,273]. A recent etiological study demonstrated that a feed-forward mechanism occurs between chronic inflammation (e.g., elevated TNF-α) and elevated activity of protein kinase glycogen synthase kinase-3 (GSK-3) in MDD. This protein’s functions have extensive implications in neurodegenerative diseases such as Alzheimer’s disease [274,275], in which neuroinflammation, together with PNN, PV+ cells, and γ activity alterations, represents core pathophysiological players [276]. Thus, it has been hypothesized that a stress-meditated cascade involving GSK-3 activation and inflammatory cytokines dysregulation promotes the development of MDD [277]. Importantly, animal model studies demonstrated that the inhibition of GSK-3 with lithium treatment modulates the γ-band activity [278], and GSK-3 inhibition ameliorates deficits in spatial WM and increases evoked γ power in the primary auditory cortex [279]. Depression-like behavior and decline in PV+ cells induced by chronic unpredicted mild stress (CUMS) in mice have been linked to neuroinflammatory dysregulation [280]. Interestingly, inducible nitric oxide synthase (iNOS) (i.e., a major downstream mediator of inflammation) appears to be upregulated in CUMS models [281,282].

### 4.2. Perineuronal Net Abnormalities in Major Depressive Disorder

Evidence for GABAergic pathology in MDD has been linked to PNN alterations and abnormal deposition, with subsequent impairment of PV+ cell activity and pyramidal cell hyperexcitability [283,284]. Interestingly, it has been proposed that the mechanisms underlying the effect of antidepressant medications may involve metalloproteinases (MMPs), which impact PNN structures around PV+ interneurons when their levels are elevated, with subsequent disruption of the E/I balance [57,285]. This appears to be the case with the serotonin and norepinephrine reuptake inhibitor *venlafaxine*, which increases hippocampal levels of MMP-9 protein and subsequently attenuates PNNs [286]. This effect can be explained by the ability of monoamine reuptake inhibitors to increase the expression of PNN-degrading proteases such as MMP-9 [287], as well as to impact γ activity [283].

Yu and colleagues [284] investigated PNN density in the prelimbic cortex in animal models of CUMS. Depressive-like behaviors emerged after CUMS, accompanied by a decrease in PNN+ cell density and aggrecan expression. Moreover, in the same study, rats were divided into two subpopulations based on the exhibited behaviors: vulnerable versus resilient. The density of PNNs and the expression level of neurocan in the vulnerable group were decreased compared to the control and resilient groups. Behavioral examination of locomotion showed that PNN density and neurocan levels were lower in rats, defined as “low responders” in the new context, compared to “high responders”. An increase in the number of PNNs surrounding PV+ interneurons has also been detected [288]. Thus, animal models of depression suggest stress-induced changes in ECM/PNNs and PV+ interneurons [289], with the direction of these alterations largely depending on stress recency and timing [290]. Specifically, a biphasic temporal regulation (i.e., both increase and decrease) of ECM/PNNs is observed after the stress [291]. In light of the above-mentioned research, there is emerging evidence for the role of PNNs in depression despite inconsistencies attributable to the specificity of different stress models.

The literature on PNN disturbances in MDD patients consists of studies on molecular alterations. Some evidence has been provided for decreased reelin expression in the PFC, hippocampus, cerebellum, and blood of MDD patients, as in BD and ScZ [69,292,293]. Furthermore, similar to ScZ, increased levels of MMP-9 have been reported in patient blood samples during depressed states [71,294]. Further studies are needed to understand the molecular underpinnings of PNN alternations in MDD. Despite the evidence from human studies being scarce, the intriguing results on the impact of treatments for mood disorders on ECM molecules and PNN composition from animal models imply a need for further investigation.

### 4.3. PV+ Interneuron Alterations in Major Depressive Disorder

Accumulating evidence suggests that the GABAergic system is altered in the brain of patients with MDD. The available molecular knowledge mainly derives from stress models [295]. For instance, CUMS leads to a reduction of GAD67 levels in both rat PFC and hippocampus, as well as in vitro cortical neurons [296]. PV+ cells also decreased in the dentate gyrus, hippocampus, and mPFC after exposure to long-term psychosocial stress [297,298]. Interestingly, electrophysiological analyses revealed reduced frequencies of spontaneous IPSCs, decreased release probability of GABAergic synapses, and alterations in GABAB receptor-mediated signaling. In turn, pyramidal neurons showed a higher excitability [299]. Kigawa and colleagues [300] also found that combined exposure to prenatal and adolescent stress reduces PV+ interneurons. Interestingly, epigenetic changes in the GABAergic system seem to be involved in stress-related models exhibiting depression-like behaviors [301]. The outlined findings from chronic stress provide strong support for a fronto-limbic PV+ involvement in the emotional and cognitive symptoms of MDD.

Clinical evidence suggests that MDD is associated with reduced levels of GABA in the plasma, cerebrospinal fluid, and neocortex [302,303,304]. Moreover, effective medications have been shown to impact the GABAergic system by reversing low levels of GABA in the occipital cortex [305,306]. The literature from human studies on PV+ alterations is, however, inconclusive. A post-mortem study investigating PV+ and CB+ cells in the DLPFC and orbitofrontal cortex (OFC) in MDD brains reported a reduction of CB+ cell density and size compared to the control group, especially in the DLPFC region, while a trend toward reduction emerged for PV+ interneurons [307]. Reduced density of CB+ cells has also been reported in patients’ somatosensory cortex, while PV+ interneurons showed a smaller reduction that did not reach statistical significance [308]. Sibille and colleagues [243] found that MDD patients have reduced SST+ mRNA expression in the DLPFC, and no alterations in other transcripts. Similarly, Zhang & Reynolds [156] reported no significant reduction in PV+ neurons compared to BD and ScZ patients.

The outlined findings from human studies provide some evidence for abnormalities in distinct subpopulations of GABAergic interneurons in MDD. However, the framework is not definitive, possibly due to methodological limitations and study population specifics (e.g., death by suicide, antidepressant medication use at the time of death). It might be argued that GABAergic abnormalities qualitatively differ to some extent among neuropsychiatric disorders, potentially in correlation with macro-level manifestations [246,309].

### 4.4. Gamma-Band Oscillatory Alterations in Major Depressive Disorder

Brain oscillatory alterations in the γ-band represent an emerging topic in MDD research [310], with evidence deriving both from animal models and patients. Deep brain stimulation (DBS) of the ventral tegmental area (VTA) in a mouse model of depression was shown to induce an increase in the high γ-band phase coherence, which was correlated with an improvement in depressive-like behavior [311]. Also, Khalid and colleagues [312] demonstrated that remission from depressive behavior induced by chronic restraint stress in mice is associated with the restoration of γ activity. Riga and colleagues [313] showed that the administration of *Vortioxetine*, a multimodal antidepressant drug with pro-cognitive actions on GABAergic signaling, modulates γ oscillations in the paraventricular thalamic nucleus and prevents recognition memory deficits. Moreover, *Vortioxetine* appears to prevent the γ coherence deficits in the prelimbic nucleus–dorsal hippocampus pathway.

Electrophysiological evidence in humans showed that rsEEG metrics for γ rhythms discriminate healthy controls from MDD patients [314] and predict behavioral responses (i.e., abnormal responses to task errors, potentially due to lower γ activity within regions subserving affective and/or motivational responses to salient cues) [315]. Also, it was found that during the maintenance phase of WM tasks, MDD patients exhibit less occipital γ and that the severity of depression correlates with this alteration [316]. Increased γ-band EEG in a sustained semantic information processing task was detected in MDD patients, while a decrease throughout the task emerged in patients with ScZ, suggesting qualitative differences in γ-band disturbances between these disorders for elaborative emotional processing (i.e., higher physiological reactivity to negative information in depressed patients) [317]. Distinct patterns and locations of oscillatory alterations have been found to distinguish MDD from other disorders during emotional tasks (e.g., γ activity in the parietal and left posterior temporal cortex as an index to differentiate BD and MDD patients during implicit emotional tasks) [318,319].

Repetitive TMS paradigms over the DLPFC in MDD have been adopted as a treatment for pharmacoresistant patients to ameliorate symptoms [320] and to restore brain network functional connectivity among nodes including the left insula, right superior and inferior frontal gyrus, right inferior parietal lobule, and amygdala [321]. Paire-pulse protocols revealed increased cortico-cortical inhibition [322], supporting the hypothesis of GABAergic tone reduction in this disorder [323]. However, evidence from brain stimulation studies on γ oscillations remains scarce. Canali and colleagues [262] reported reduced TMS-evoked frontal γ-band frequencies after TMS over the premotor area in MDD patients (as well as BD and ScZ) compared to healthy subjects. Another study showed that two weeks of high-frequency rTMS over the left DLPFC of MDD patients determines an increase in frontal resting γ power and central-temporal θ-γ coupling at electrode sites. Furthermore, increased frontal γ power correlated with improved scores on the Hamilton Rating Scale for Depression (HAM-D17) and Beck Depression Inventory (BDI), while the increased central coupling correlated with a reduced number of errors on the Wisconsin Card Sorting Test (WCST) [324]. These findings are not in line with a recent rTMS study in which increased anterior-central γ power correlated with worsened depressive symptoms. These inconsistencies might be attributed to the complexity of γ oscillation dynamics over the course of TMS, as well as to individual differences in brain responses [325]. As such, MDD might be characterized by either elevated or decreased γ oscillation power, with varying values of “optimal control” among patients [310].

**Figure 4:**
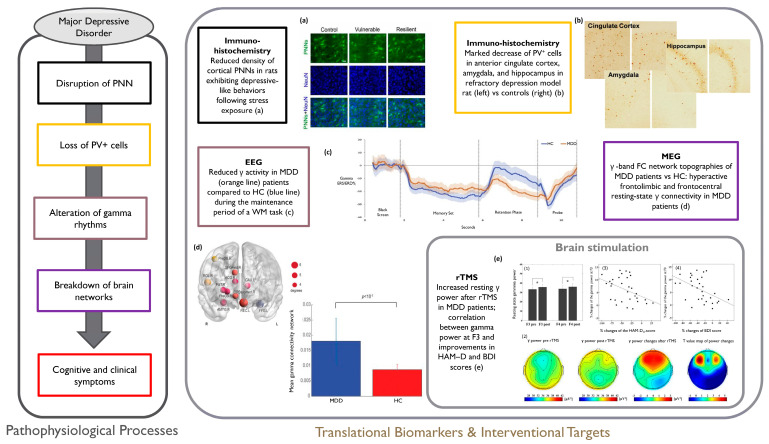
Overview of the outlined pathophysiological cascade in MDD (**left panel**) with relevant sample evidence (**right panel**). (**a**) Representative images of immunofluorescence staining of PNNs and quantification of neurons (neuronal marker: NeuN+) in the PrL of HC compared to vulnerable and resilient rats in response to CUMS (Reprinted from [284] (licensed under CCBY 4.0, https://creativecommons.org/licenses/by/4.0/, accessed on 7 December 2023); (**b**) Decreased number of PV+ cells in coronal sections of the cingulate cortex, amygdala and hippocampus in HC vs. refractory depressed rats (Reprinted from [300] (licensed under CCBY 4.0, https://creativecommons.org/licenses/by/4.0/, accessed on 7 December 2023); (**c**) Graph for modulation of γ oscillatory power calculated as ERS/ERD% (with data pooled across O1 and O2 electrodes): positive values reflect ERS, negative values reflect ERD. Gamma ERS/ERD% during the Sternberg task was lower for MDD patients (orange) compared to HC (blue) (Reprinted from [316] with permission from Elsevier)); (**d**) Hyperactive frontolimbic and frontocentral resting-state γ connectivity in MDD: γ-band functional connectivity network differences when comparing MDD patients to HC at rest: top 11 most connected brain regions with node sizes representing the number of connections within the network (**upper left**), grand average of γ functional connectivity strengths within the identified network across MDD patients and HC, respectively (**bottom right**) (Reprinted from [327] with permission from Elsevier); (**e**) Increased γ power in MDD patients following rTMS: (**e,1**) Resting γ power: bar graphs showing mean resting γ power before and after rTMS at the F3 and F4 electrode sites across MDD patients (* indicates significance); (**e,2**) EEG topographical plots of resting-state γ power; from right to left, the topoplots depict the γ power distribution pre-rTMS, post-rTMS, the difference between pre- and post-rTMS, and the t-value map corresponding to the difference between pre- and post-rTMS; (**e,3,4**) Scatter plots showing positive correlation between the resting γ power increase at the F3 electrode site and improvements in HAM-D17 and BDI (Reprinted from [324] with permission from Elsevier). PNN = Perineuronal Net; HC = Healthy Controls; PrL = Prelimbic cortex; CUMS = Chronic Unpredictable Mild Stress; PV+ = Parvalbumin-positive; ERS = Event-Related Synchronization; ERD = Event-Related Desynchronization; ERS/ERD% = Event-Related Synchronization/Desynchronization Percentage; MDD = Major Depressive Disorder; rTMS = repetitive Transcranial Magnetic Stimulation; EEG = Electroencephalography; HAM-D17 = Hamilton Depression Rating Scale-17; BDI = Beck Depression Inventory.

The outlined literature provides some evidence for γ-band abnormalities as a relevant feature, as well as a potential biomarker and therapeutic target for DBS and NIBS treatments in MDD [326]. Further research is needed to clarify the dynamics and patterns of γ alterations in relation to clinical manifestations. See Figure 4 for an overview of the outlined framework in MDD.

## 5. Discussion

### 5.1. Gamma-Band-Related Pathophysiology

Alterations in brain oscillations represent a core aspect of different psychiatric disorders [328]. Rhythmic disturbances in ScZ have been extensively reported and reviewed, with γ oscillations recognized as a substrate of both clinical symptoms and cognitive impairments [329]. Furthermore, the processes underlying this physiological dysfunction could be incorporated into a pathophysiological framework summarized as follows. Gamma-band alterations result from an imbalance between excitation and inhibition in the brain [330], particularly from weakened PV+ cells’ inhibitory inputs onto the pyramidal neurons [142]. Cortical hyperexcitability and interneuron dysfunction have also been associated with PNN impairment [57], which disrupts interneuronal plasticity and creates the environment for chronic neuroinflammation, with detrimental effects on PV+ cells [65,331]. Similar proofs for γ alterations and the underlying pathophysiology have been reported in BD, and growing evidence corroborates this cascade in MDD. Both rodent and human studies support the delineated multi-dimensional explanation of brain dysfunction from PNN environment disruption to PV+ alterations and oscillatory disturbances.

The evidence for the involvement of GABAergic interneurons mainly involves PV+ cells, which represent the main source of γ oscillation [38], both in microcircuits [43] and at the network level [41]. However, evidence for CB+ and SST+ interneurons dysfunction and/or reduction is also available from studies on ScZ, BD, and particularly MDD [242,245]. Noteworthy, some studies have shown that the activity of non-PV+ GABAergic interneurons may also be involved in fast oscillations, particularly when considering the network activity [332]. Despite the evidence for a prominent role of PV+ interneurons in γ activity, an unequivocal link between specific cell types and the production of rhythms at a certain frequency would be simplistic. For instance, FS bistratified cells exhibit a strong coupling with the hippocampal γ oscillations cycle [333]. SST+ interneurons seem to be involved in γ rhythms, as demonstrated by the decrease in the power of slow γ oscillations following SST+ cell disruption, as for PV+ cells. Moreover, photoexcitation on SST+ interneurons seems to generate γ oscillations. Indeed, complementary roles for SST+ and PV+ interneurons have been hypothesized in the generation of γ rhythms [157]. At the neurochemical level, SST+ and CB+ cells exhibit features predominantly attributed to PV+ neurons (i.e., expression of the voltage-gated K+ channel subunits Kv3, characterized by short and rapid currents and immunoreactivity to PNN markers) [334,335,336]. This suggests that some non-PV neurons may exhibit fast-firing properties [68].

The second aspect worthy of discussion is represented by the association of GABAergic with glutamatergic abnormalities in determining the E/I imbalance [20]. A strong correlation emerges between levels of glutamate+glutamine (Glx) and GABA from magnetic resonance spectroscopy (MRS) databases [337]. Glutamatergic medications (i.e., *Ketamine*) potentiate GABAergic synapses by blocking NMDA-R located on GABA neurons [271,338]. As introduced earlier, the E/I imbalance causing impaired γ rhythm stems from glutamate and GABA coexistent dysfunction and has been robustly reported in psychiatric and neurological disorders [32,34,339]. Altered levels of Glx, reduced pyramidal cell density, and attenuated NMDAR neurotransmission characterize ScZ, with differences depending on disease stage and treatment history [340,341]. Importantly, a reduction and/or hypo-activity of NMDAR has been documented and linked to GABAergic circuits dysfunction [342]. Indeed, NMDAR impairments generate abnormalities in levels of GAD67 in PV+ interneurons, as well as in PV expression [140,196], supporting the role of NMDAR-mediated glutamatergic signaling in GABAergic interneuronal development and behavior [343]. Aberrant glutamatergic modulation of GABA interneurons is also evident in BD, in which abnormal levels of Glx [344] and increased cortical glutamate levels [235] have been detected. Consistent with the GABAergic-glutamatergic modulation of GABA neurotransmission, Woo and colleagues [345] showed a decreased density of GAD67 mRNA–containing neurons in BD patients and an even higher reduction of cells co-expressing the NMDA NR(2A) subunit [346]. Also, MDD patients exhibit increased levels of glutamate and altered NMDARs function in the PFC, which has been specifically linked to altered DMN connectivity [347,348]. Crucially, it has been hypothesized that GABAergic deficit–induced changes in neural excitability and reduced conversion of glutamate to GABA may be responsible for the increased glutamate concentrations found in the brain of psychiatric patients [349]. However, given the evidence for reduced rather than increased brain content of glutamate in psychiatric patients [350], the relationship between GABAergic and glutamatergic transmission changes needs to be further elucidated.

### 5.2. Evidence of Gamma-Band-Related Pathophysiology in Other Mental Disorders

The literature supporting the pathophysiological framework outlined in this narrative review suggests that γ abnormality and its underlying cellular and molecular alterations are abundant in psychosis and mood disorders. Some pieces of evidence on psychiatric conditions other than ScZ, BD, and MDD can be mentioned to corroborate the relevance of the delineated framework in psychiatry. For instance, recent work from Briones and colleagues [351] revealed PV+ cells and their surrounding PNNs to be altered in the dorsal striatum of mice models of excessive repetitive behavior (i.e., relevant for Obsessive-compulsive Disorder and other neuropsychiatric disorders). Interestingly, this was linked to abnormalities in inhibitory postsynaptic potentials, reduced γ power, and altered dendritic spine density, with normalization of repetitive behavior following the restoration of PNNs and PV+ cells. Given the heterogeneity of the presented literature, particularly in terms of methodological variations across studies (e.g., immunolabeling techniques, mice model types, medications in patients’ samples), caution should be applied when interpreting and linking results. This also points to the need for further investigation of the outlined pathophysiological framework.

A relevant point that should be discussed is the MDD evidence, abundantly relying on mice models of stress (e.g., CUMS) [284]. Although the outlined literature refers to depression-like behaviors [301] and their link with γ oscillations [313], results from these models might be relevant for a wide span of psychiatric conditions, including generalized anxiety, panic, social and separation anxiety, agoraphobia, post-traumatic stress, and OCD [352]. Moreover, early life adversity events, that is, the case of environmental stress models, have been linked to the development of both anxiety disorders and clinical depression [353]. Interestingly, evidence for the involvement of PV+ interneurons in anxiety emerged from a mouse model of mutant Erbin (i.e., a protein expressed in cortical and hippocampal PV+ interneurons regulating fast excitatory synaptic transmission) [354], with inhibition of PV+ cells resulting in anxiogenic effects and reduced excitatory postsynaptic responses in the amygdala [355]). Further studies are needed to elucidate the pathophysiology of PV-related γ dysfunction in different stress-related disorders.

Deficient neural oscillatory patterns in the γ frequency band have also been reported in Autism Spectrum Disorder, together with evidence for a reduction in the PV+ expression [356,357] as well as PNN alterations in mice models [358,359]. Neuroinflammation has also been detected and associated with reduced GABA levels [360,361]. Given the recent evidence for the outlined pathophysiology involving PNNs and PV+ interneurons, neural oscillations in the γ band are receiving growing attention as a potential target in Autism Spectrum Disorder [34,362]. The outlined evidence corroborates the relevance of the γ-related pathophysiology in psychiatry as a valuable topic for further investigation of disease biomarkers and the potential development of new therapeutics.

The pathophysiological cascade outlined in this review has recently gained attention also in the field of dementia, particularly Alzheimer’s Disease [363,364]. Indeed, degradation of PNN and neuroinflammation have been reported in multiple neurodegenerative diseases, as well as E/I imbalance and hyperexcitability subserving γ-band oscillatory alterations [25,365,366]. These have been attributed to PV+ interneurons’ dysfunction [367,368], which seems to also increase brain vulnerability to β-amyloid (Aβ) deposition [369]. Accordingly, a growing body of research is currently investigating the therapeutic effects of γ-induction via NIBS or multisensory stimulation on multiple pathological facets of neurodegeneration. Promising results emerge from both animal models [370,371,372] and studies on patients (i.e., behavioural improvements, restoration of intracortical connectivity, increased brain perfusion, and reduced tau burden) [373,374,375,376,377].

### 5.3. Conclusions: Brain Oscillatory Biomarkers and Non-Invasive Treatments

In his pioneering work, *Rhythms of the Brain*, Buzsáky remarked that

‘[…] gamma oscillations, and neuronal oscillations in general, are not “independent” events that impose timing on neuronal spikes but rather are a reflection of self-organized interactions of those neurons’.

[378]

Investigating the molecular underpinnings of altered brain oscillations offers great insights into disease mechanisms involving these disturbances. Given the heterogeneity and complexity of psychiatric disorders, this might support the search for valuable biomarkers, which represents one of the major challenges in the field of precision medicine. Indeed, oscillatory alterations, as in the γ rhythms, offer a dynamic picture of brain disturbances, even before the full development of the disorder is evident [328]. In this regard, MEG and EEG-based research has attracted increased interest in the identification of markers for neuropsychiatric disorders [379]. We believe two reasons delineated this scenario. First, abnormal brain oscillations have the potential to be adopted as biomarkers [380]. Secondly, the recent combination of EEG with NIBS allows oscillatory activity and other cortical properties underlying brain rhythms (e.g., excitability, inhibition, connectivity, and plasticity) to be directly modulated and causally investigated [12]. As already mentioned, the TMS-EEG methodology has shown its potential to uncover biological targets in psychiatric illnesses and, not least, to advance our understanding of biological, physiological, and clinical features integrated into a comprehensive pathological framework [381].

As also shown in the field of dementia [382], abundant evidence from the last two decades points to the use of NIBS as a promising avenue for developing effective interventions in psychiatry [383]. As such, rTMS is already an established interventional technique for MDD, and further evidence is available in BD and ScZ, especially for the treatment of hallucinations, which have also been treated with tDCS (See [384] for an overview of NIBS treatments in psychiatric disorders) [385]. Promising results have recently emerged from clinical trials adopting tACS for the restoration of disturbed brain oscillations in ScZ [386] and MDD [387]. In addition to its potential therapeutic role, brain responses to tACS could serve as a possible tool for determining the diagnosis, classification, and prognosis of psychiatric disorders [388,389]. Further methodological development of neuromodulation targeting brain oscillations benefits from the elucidation of the pathophysiology of illness, from physiological alteration to its neurobiological underpinnings.

Future studies would further elucidate the presented framework and provide evidence for causal relationships between common pathophysiological factors across psychiatric disorders. Indeed, potential confounding factors (e.g., medication effects, substance use, comorbidities) on the presented neuropathological framework should be further addressed together with the impact of genetic predisposition and environmental influences.
